# Selenium-Binding Protein 1-Deficient Dendritic Cells Protect Mice from Sepsis by Increased Treg/Th17

**DOI:** 10.3390/antiox14040468

**Published:** 2025-04-14

**Authors:** Xin Zhang, Shuang Han, Zhu Zeng, Jie Dai, Yi Jia

**Affiliations:** 1 Key Laboratory of Infectious Immune and Antibody Engineering of Guizhou Province, Cellular Immunotherapy Engineering Research Center of Guizhou Province, School of Basic Medical Sciences/School of Biology and Engineering (School of Modern Industry for Health and Medicine), Guizhou Medical University, Guiyang 550025, China; zhangxin@stu.gmc.edu.cn (X.Z.); hanshuang@stu.gmc.edu.cn (S.H.); zengzhu@gmc.edu.cn (Z.Z.); 2 Immune Cells and Antibody Engineering Research Center of Guizhou Province, Key Laboratory of Biology and Medical Engineering, Guizhou Medical University, Guiyang 550025, China

**Keywords:** selenium binding protein 1, dendritic cell, redox imbalance, sepsis

## Abstract

Selenium-binding protein 1 (SELENBP1) has been implicated in cancer development, neurological disorders, tissue injury, metabolic regulation, and cell differentiation. Sepsis is characterized prominently by immunological dysregulation and severe organ damage. However, whether SELENBP1 improves sepsis by regulating immune cell activity remains unknown. Here, we detected an elevation of SELENBP1 levels in the blood of sepsis patients and in the livers of septic mice. Significantly, SELENBP1 knockout (KO) prolonged survival in septic mice. This phenomenon was accompanied by decreased liver damage, reduced inflammation levels, and an increased regulatory T cell/T helper 17 cell (Treg/Th17) ratio in the spleen. Additionally, SELENBP1 deficiency induced a redox imbalance and inhibited dendritic cell (DC) maturation, resulting in a tolerogenic DC (tolDC) phenotype and an increase in the Treg/Th17 ratio. Furthermore, SELENBP1-KO mature DCs (mDCs) alleviated liver injury by increasing the Treg/Th17 ratio in the spleen, thus improving the survival of septic mice. These findings indicate that SELENBP1 is involved in sepsis by regulating DC immune activity, which might provide a potential way for sepsis treatment.

## 1. Introduction

Selenium-binding protein 1 (SELENBP1), found in bacteria, plants, and mammals [1], is involved in selenium metabolism via direct selenium binding [2,3,4] or indirect selenium delivery through interactions with selenophosphate synthetase [5], glutathione peroxidase 1 (GPx1) [6], or selenoprotien P (SELENOP) [7]. SELENBP1 functions as a copper-dependent methanethiol oxidase that degrades sulfur-containing chemicals associated with halitosis [8,9]. Additionally, it contributes to heavy metal detoxification through an unknown mechanism [10,11,12]. Furthermore, SELENBP1 has been shown to inhibit the progression of colon and prostate cancer via glucose/lipid metabolism [13] and energy metabolism [14]; it has also been linked to the survival of various cancers [2,3,13,14,15]. Moreover, SELENBP1 has been identified as a biomarker for kidney injury [2,10], cardiovascular disease [16,17,18], and neurological disorders [19]. Notably, SELENBP1 deficiency protected nematodes from oxidative stress and increased their longevity [20], but it also made them more sensitive to selenium toxicity [21]. In mice models, reduced levels of SELENBP1 ameliorated oxidative stress-induced colitis [22]. However, mice lacking SELENBP1 exhibited heightened susceptibility to oxidative stress-induced nerve injury within the hippocampus [19], underscoring its essential role in regulating oxidative stress. Moreover, SELENBP1 is involved in the differentiation of adipocytes [23] and colonocytes [24,25]. Overall, while SELENBP1 performs a range of biological functions, the detailed mechanisms underlying these actions remain inadequately understood.

Previous research has indicated that septic patients with lower urine levels of SELENBP1 have a higher likelihood of survival [26]. However, the role and mechanism of SELENBP1 in sepsis remain unclear. Sepsis is characterized by redox imbalances, excessive inflammation, and immunological dysfunction. Antioxidant and immunomodulatory therapy have been shown to enhance survival rates in septic patients [27,28,29,30]. Additionally, a high Treg/Th17 (regulatory T cell/T helper 17 cell) ratio is related to a better prognosis in septic individuals [28], implying a therapeutic role for Treg/Th17 regulation in sepsis [31]. Dendritic cells (DCs) include immature DCs (imDCs), semimature DCs (smDCs), and mature DCs (mDCs). Both smDCs and regulatory DCs (DCregs) are capable of promoting Treg differentiation while exhibiting tolerogenic properties characteristic of tolerogenic DCs (tolDCs) [32,33,34]. Notably, smDCs and tolDCs may serve as components of immunotherapeutic strategies for sepsis [35]. Furthermore, oxidative stress regulates immune cell activity, including DCs, since reactive oxygen species (ROS) are essential for the differentiation and antigen presentation of DCs [36,37]. Previous reports have revealed that SELENBP1 regulates oxidative stress responses [19,20,21,22], and bioinformatics analyses have revealed its influence on immune infiltration within colon cancer, such as through DCs and T cells [14]. Recent reports indicate that selenoproteins like selenoprotein K and methionine sulfoxide reductase B1 contribute to the immunological activity of DCs [38,39,40,41,42,43,44,45,46]. However, whether SELENBP1 effects sepsis by regulating DCs’ function via oxidative stress remains unexplored.

We hypothesized that SELENBP1 could regulate the immune function of DCs through oxidative stress, potentially leading to the generation of tolDC-like phenotypes suitable for sepsis treatment. We therefore assessed SELENBP1 levels in blood samples from sepsis patients and in liver tissues from septic mice. Subsequently, we investigated survival, liver damage, inflammation, and the immunological microenvironment of the spleen in LPS-challenged SELENBP1-deficient mice. Next, the impact of SELENBP1 deletion on the immunological function of DCs was investigated in vitro, and the efficacy of SELENBP1-deficient DC transfusion in sepsis-infected mice was evaluated. Our results describe the immunological involvement of SELENBP1 in sepsis and demonstrate the potential of SELENBP1-deficient DCs as a treatment for sepsis.

## 2. Materials and Methods

### 2.1. Reagents

Lipopolysaccharide (LPS) was purchased from Sigma (St Louis, MO, USA). Fetal bovine serum (FBS) and RPMI 1640 medium were obtained from Gibco (New York, NY, USA). Recombinant murine granulocyte macrophage colony stimulating factor (rmGM-CSF), recombinant murine interleukin-4 (rmIL-4), chemokine (C-C motif) ligand 5 (CCL5), CCL19, and FITC-Dextrans were purchased from Peprotech (Cranbury, NJ, USA). PerCP-Cyanine5.5-CD11c, FITC-CD40, FITC-CD80, FITC-CD86, APC-MHC-II, PE-CCR5, PE-CCR7, BV421-CD205, FITC-CD4, PE-Foxp3, and APC-IL-17A were obtained from eBioscience (San Diego, CA, USA). A naïve CD4^+^ T cell isolation kit was obtained from Miltenyi (Bergisch Gladbach, Germany). The Elisa kits of interleukin 1β (IL-1β), IL-6, IL-10, IL-12p70, tumor necrosis factor-α (TNF-α), and transforming growth factor-β (TGF-β) were obtained from ThermoFisher (Waltham, MA, USA). Tert-Butyl hydroperoxide (T-BHP) was obtained from Aladdin (Shanghai, China). ROS GreenTM H_2_O_2_ Probe was purchased from Maokang (Shanghai, China). The ROS assay kit, glutathione (GSH) and glutathione disulfide (GSSG) assay kit, malondialdehyde (MDA) content assay kit, superoxide dismutase (SOD), catalase (CAT), GPx activity assay kit, CCK8, phosphate buffer saline (PBS), Annexin V-FITC/PI kit, and whole blood protein extraction kit were purchased from Solarbio (Beijing, China). GAPDH antibody and SELENBP1 antibody were purchased from HUABIO (Hangzhou, China) and Abcam (Cambridge, UK), respectively.

### 2.2. SELENBP1 Analysis in Public Databases and Blood from Sepsis Patients

Transcriptomic data from the whole blood of healthy and sepsis patients were downloaded from https://www.ebi.ac.uk/biostudies/arrayexpress/studies (accessed on 23 December 2022) (E-MTAB-5273: 10 healthy; 221 sepsis patients) and the Gene Expression Omnibus (GEO) database (GSE65682: 42 healthy and 685 sepsis patients; GSE185263: 44 healthy and 348 sepsis patients). *SELENBP1* levels were then analyzed, and patient survival was calculated based on low and high *SELENBP1* expression using the quartile method. Survival curves for patients in the GSE65682 dataset were plotted. In addition, the GSE10246 dataset was used to evaluate *SELENBP1* expression in mouse tissues and immune cells. Blood samples from healthy controls (n = 4; 3 males and 1 female; age: 47 ± 4.16) and sepsis patients (n = 4; 4 males; age: 56.5 ± 16.66) were collected at QianNan People’s Hospital. The diagnostic criteria for sepsis were T/CRHA 022–2023, and the blood samples were collected from untreated patients after diagnosis. The whole research process was approved by the Ethics Committee of Guizhou Medical University, with informed consent obtained.

### 2.3. Mice

*SELENBP1* heterozygote mice (C57BL/6) were generated by Saiye Biotechnology Co., Ltd. (Suzhou, China) using the CRISPR/Cas method. gRNA was designed to delete the region from exons 1–12. PCR amplification was performed using DNA isolated from mouse tails with the following primers: Primer-F, CGCTGCATACCTGAGAGTGACTTTG; Primer-WT/He-F, GTCTACTATGTTCAGGGTTCTCTGCTTGTTT; Primer-R, GCTCGGAACTACTTTCTATTTGTGATCTCC. The proteins prepared from kidney were assayed by Western blotting to confirm successful knockout of SELENBP1. Homozygous mice (knock out, KO) and wild-type (WT) mice generated from heterozygous mice were used for experiments. All animal studies comply with the guidelines of the Experimental Animal Ethics Committee of Guizhou Medical University (No. 2100275).

### 2.4. Septic Shock Model

Eight-week-old C57BL/6 mice were divided into four groups: WT + PBS, KO + PBS, WT + LPS, and KO + LPS, with 20 mice for each group. The mice fasted for 12 h before intraperitoneal injection and drank freely. Following the administration of 25 mg/kg LPS via intraperitoneal injection, feed and water were administered, after which the survival probability was evaluated.

### 2.5. Septic Model

The 8-week-old C57BL/6 mice were divided into four groups: WT + PBS, KO + PBS, WT + LPS, and KO + LPS, with 20 mice for each group. The animals fasted for 12 h before intraperitoneal injection and drank freely. A total of 10 mg/kg LPS was injected into the mice intraperitoneally, and 4 h later, some mice were anesthetized and killed for collection of blood, ascites, and spleen in order to evaluate immune cell composition and inflammatory cytokines. Additionally, livers and spleens from other subjects were collected for subsequent experiments after 12 h of LPS treatment.

### 2.6. HE Staining

The livers from septic mice challenged by LPS were washed with physiological saline and fixed overnight in paraformaldehyde before undergoing dehydration with a gradient of ethanol, followed by xylene transparency and paraffin embedding. After deparaffinization, HE staining was performed. In brief, the dewaxed sections were stained with a hematoxylin solution followed by washing with water; then, they underwent hydrochloric acid–ethanol and ammonium hydroxide treatments. The slices were stained with eosin solution, dehydrated, transparent, sealed with neutral gum, and photographed using a Nikon microscope (Tokyo, Japan).

### 2.7. Spleen Immunophenotype Assay

The spleens of septic mice were collected, homogenized, and prepared as cell suspensions. The cells were then treated with red blood cell lysate followed by washing with PBS. DCs cells were stained using CD11c, CD40, CD86, or MHC-II after fixation. Tregs and Th17 cells were stained with CD4/Foxp3 and CD4/IL-17A, respectively, following fixation/permeabilization procedures. After washing with PBS, the cells were subjected to flow cytometry (BD, Biosciences, Franklin Lakes, NJ, USA), and then the DCs counts and Treg/Th17 ratio were calculated using FlowJo (V10, BD, Biosciences).

### 2.8. Immune Cell Detection in the Blood and Ascites of Septic Mice

The blood and ascites from septic mice were collected and analyzed using an animal automatic blood cell analyzer (Mindray, Shenzhen, China) capable of detecting various body fluids, including ascites. The white blood cells (WBC), neutrophils, monocytes, and lymphocytes were then counted and analyzed.

### 2.9. DCs Preparation and Phenotypic Detection

Bone marrow from WT and *SELENBP1* KO mice was collected and treated with red blood cell lysate, followed by washing with RPMI 1640 medium containing 1% FBS. The cells were cultured in an incubator at 37 °C with 5% CO_2_ for seven days in RPMI 1640 medium with 10% FBS, 20 ng/mL rmGM-CSF, and 10 ng/mL rmIL-4 to prepare imDCs. mDCs were then prepared by adding 100 ng/mL LPS for 1 day [39,40]. The DCs were collected, washed with PBS, and fixed with 4% paraformaldehyde for 15 min. Following a wash with PBS, the cells were incubated in the dark at 4 °C for 20 min with a 1% BSA solution of antibodies to CD11c, CD40, CD80, CD86, and MHC-II, followed by washing and suspension with PBS. Then, the DCs were subjected to flow cytometry and analyzed with FlowJo.

### 2.10. Cell Viability and Apoptosis Assay

A total of 1 × 10^6^/mL imDCs or mDCs were plated into a 96-well plate; after adding CCK8 reagent, they were incubated in an incubator for 1 h at 37 °C in 5% CO_2_ conditions. Then, the absorbance was measured using a microplate reader at 450 nm to assess cell viability. Apoptosis of DCs was evaluated using an Annexin V-FITC/PI kit. In brief, 1 × 10^5^ imDCs or mDCs were collected and incubated with 10 μL Annexin V-FITC for 15 min in the dark. Flow cytometry was performed after adding 5 μL PI for an additional 5 min, and apoptosis was analyzed using FlowJo.

### 2.11. Migration Assay

A total of 1 × 10^5^ imDCs or mDCs were placed into the upper chamber of the Transwell, while RPMI 1640 medium supplemented with 10% FBS was added to the lower chamber. After incubation for 24 h at 37 °C in a 5% CO_2_ incubator, cell counts from the lower chamber were obtained using a cell counter, and the free migration of imDCs or mDCs was analyzed. For chemotactic migration, chemokine CCL5 (50 ng/mL) was added to the bottom chamber for imDCs, while chemokine CCL19 (100 ng/mL) was used for mDCs. After 24 h of incubation in an incubator, cells in the lower compartment were counted, and the chemotactic migration was analyzed. imDCs and mDCs were stained with CCR5 and CCR7, respectively, and detected using flow cytometry [39,40].

### 2.12. Phagocytosis Assay

A total of 5 × 10^5^ imDCs were added to a 24-well plate and incubated with 1 mg/mL FITC-dextran for 0.5 h at 37 °C in a 5% CO_2_ incubator. The imDCs were then fixed with paraformaldehyde and washed with PBS. Positive cells of FITC and CD205-stained imDCs were detected using flow cytometry and analyzed with FlowJo [39,40].

### 2.13. mDCs-Induced Differentiation of CD4^+^ T Cells

Naïve CD4^+^ T cells were isolated from mouse spleens using a naïve CD4^+^ T cell isolation kit. mDCs were co-cultured with naïve CD4^+^ T cells at a ratio of 1:1, 1:10, and 1:100 for 72 h in an incubator. Subsequently, T cell proliferation was measured using the CCK8 method via microplate reader at 450 nm. In addition, naïve CD4^+^ T cells induced by mDCs (1:1) for 72 h were stained with CD4, Foxp3, and IL-17A after treatment with fixation/permeabilization solution and subjected to flow cytometry. Then, CD4^+^ T cells, CD4^+^Foxp3^+^ Tregs, and CD4^+^IL-17A^+^ Th17 cells were counted using the FlowJo.

### 2.14. Redox Status Assay

The livers from septic mice and the imDCs and mDCs prepared from WT and *SELENBP1* KO mice were collected to assess redox status. The contents of GSH and MDA as well as the activity of SOD, GPx, and CAT in the livers were assayed using corresponding kits as described by the manufacturer. Furthermore, ROS and H_2_O_2_ levels and GSH and GSSG contents along with GPx/CAT activity in DCs also underwent assessment according to manufacturer guidelines.

### 2.15. T-BHP Treatment

T-BHP was used as a ROS donor to evaluate the role of ROS in SELENBP1-deficient DCs. The cytotoxicity of t-BHP (90, 110 μM) was assayed by the CCK8 method, and a dose without cytotoxicity was selected for further experimentation. The phagocytosis and mDCs-induced differentiation of CD4^+^ T cells were assayed using the methods described above.

### 2.16. Cytokine Detection

The serum and spleen from septic mice and the supernatants of imDCs and mDCs were collected. The levels of IL-1β, IL-6, IL-12, and TNF-α in the serum, as well as the levels of IL-1β, IL-6, IL-10, IL-12, TNF-α, and TGF-β secreted by imDCs and mDCs were assessed using the manufacturer’s Elisa kits.

### 2.17. RT-PCR

Bone marrow, imDCs, and mDCs from WT mice were collected, and the RNA was extracted using Trizol. *SELENBP1* mRNA levels were evaluated by RT-PCR after reverse transcription and analyzed using the 2^−∆∆CT^ method [47]. The primers for *GAPDH* [48]: Primer-F, TGACATCAAGAAGGTGGTGAAGC; Primer-R, CCCTGTTGCTGTAGCCGTATTC. For *SELENBP1* [23]: Primer-F, TGAGCCTCTGCTCGTTCC; Primer-R, TGGACCACACTTTGTGCATT.

### 2.18. Sepsis Treatment

WT-mDCs and KO-mDCs were prepared using the methods described above. Subsequently, eight-week-old WT C57BL/6 mice were treated with 15 mg/kg LPS, followed by intraperitoneal injection with PBS, 5 × 10^6^ WT-mDCs, or KO-mDCs, and the survival probability was observed. In addition, the liver histological morphology, Treg/Th17 ratio in the spleen, and SELENBP1 levels in the liver and blood were also evaluated.

### 2.19. Western Blotting

Proteins from the liver, kidney, lung, and spleen of WT mice, as well as livers of septic mice along with imDCs/mDCs derived from WT and SELENBP1 KO mice were prepared with RIPA lysates containing protease inhibitors. The blood proteins of healthy control, sepsis patients, and septic mice were extracted by the whole blood protein extraction kit. After quantification with the BCA kit, the proteins were separated on a 10% polyacrylamide gel and then transferred to a PVDF membrane. Following a block with skim milk, the membrane was incubated overnight with SELENBP1 and GAPDH antibodies, followed by incubation with HRP-labeled secondary antibodies. After incubation with the ECL luminescent solution, SELENBP1 levels were observed by the imaging system (BIO-RAD, Hercules, CA, USA) and calculated using ImageJ (V1.48).

### 2.20. Statistical Analysis

All the experiments were repeated at least three times. The data were presented as mean ± standard deviations and analyzed using the Student’s *t*-test or analysis of variance for two or multiple group comparisons via GraphPad Prism 8.0. A *p* value of less than 0.05 was considered to indicate significance.

## 3. Results

### 3.1. Lower SELENBP1 Expression Was Associated with Higher Survival in Sepsis

**Figure 1:**
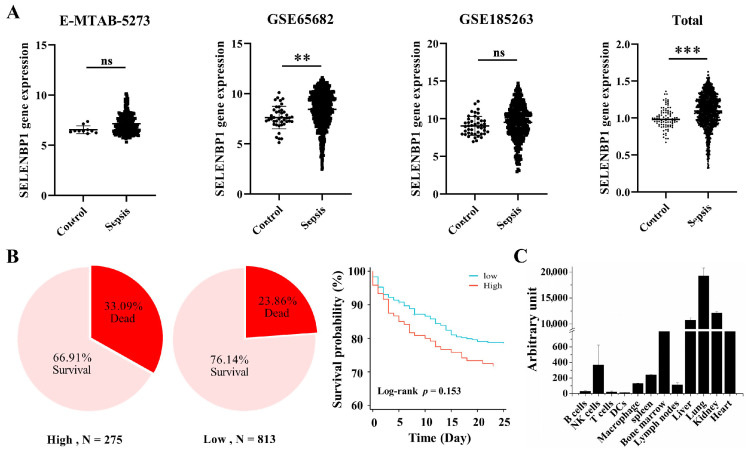
*SELENBP1* levels in sepsis patients, mouse tissue, and immune cells. (**A**) *SELENBP1* levels in the whole blood genomic datasets of sepsis patients. (**B**) The quartile method was used to evaluate survival in sepsis patients with low and high *SELENBP1* levels, and the survival probability was calculated using the GSE65682 dataset. (**C**) *SELENBP1* levels in mouse tissues and immune cells of the GSE10246 dataset. ** *p* < 0.01; *** *p* < 0.001; ns indicates no significance. SELENBP1, Selenium-binding protein 1.

**Figure 2:**
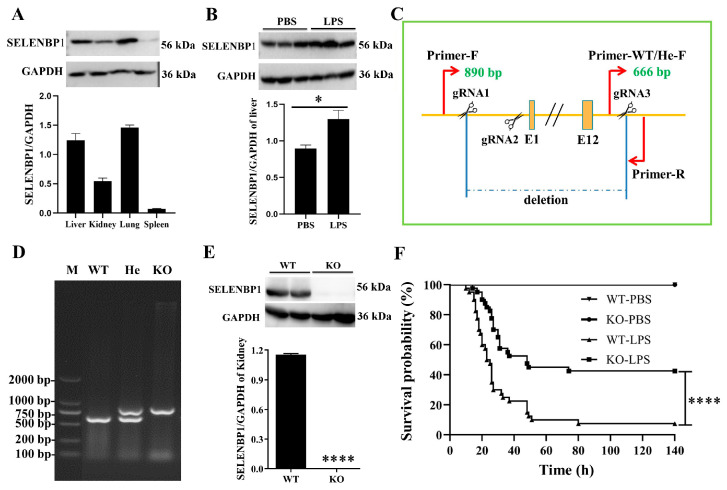
SELENBP1 was associated with survival in sepsis. (**A**) SELENBP1 protein levels in the liver, kidney, lung, and spleen of mice were detected using Western blotting (n = 3). (**B**) SELENBP1 protein levels in the livers of mice with LPS-induced sepsis (n = 3). (**C**) *SELENBP1* KO mice were generated using the gRNA targeting exons 1–12 and verified using DNA from mice tails (**D**) and proteins of the kidney (**E**). (**F**) Survival probability of septic shock model mice treated with 25 mg/kg LPS (n = 20). * *p* < 0.05; **** *p* < 0.0001; ns indicates no significance. PBS, phosphate buffer saline; LPS, Lipopolysaccharide; WT, wild-type; KO, knock out; He, heterozygous.

A previous clinical investigation revealed that elevated urine SELENBP1 levels were associated with poorer outcomes in patients with sepsis [26]. However, further studies are needed to validate the reliability of SELENBP1 as a biomarker for sepsis. In this study, we used the GEO database to examine *SELENBP1* expression in whole blood and discovered that its levels were significantly higher in sepsis patients (Figure 1A). Patients with low *SELENBP1* expression exhibited a higher survival rate compared to those with high *SELENBP1* expression in the overall dataset. Furthermore, the GSE65682 dataset indicated a better survival rate in patients with low *SELENBP1* expression (Figure 1B). Subsequently, we analyzed *SELENBP1* expression in various organs and immune cells using the GSE10246 dataset (Figure 1C). Similarly, the mice had higher levels in the liver and lung but lower levels in the kidney and spleen (Figure 2A, Supplementary Figure S1A). In addition, the livers of LPS-induced sepsis mice exhibited dramatically increased SELENBP1 levels (Figure 2B, Supplementary Figure S1B). Following this, we generated *SELENBP1*-KO mice, and genotype and protein detection confirmed successful construction of *SELENBP1*-KO mice (Figure 2C–E, Supplementary Figure S1C), and LPS-challenged *SELENBP1*-KO mice survived considerably longer than that of WT mice (Figure 2F). This indicated that decreased SELENBP1 expression was associated with enhanced survival rates during sepsis.

### 3.2. LPS-Challenged SELENBP1-KO Mice Had a Minor Liver Injury

**Figure 3:**
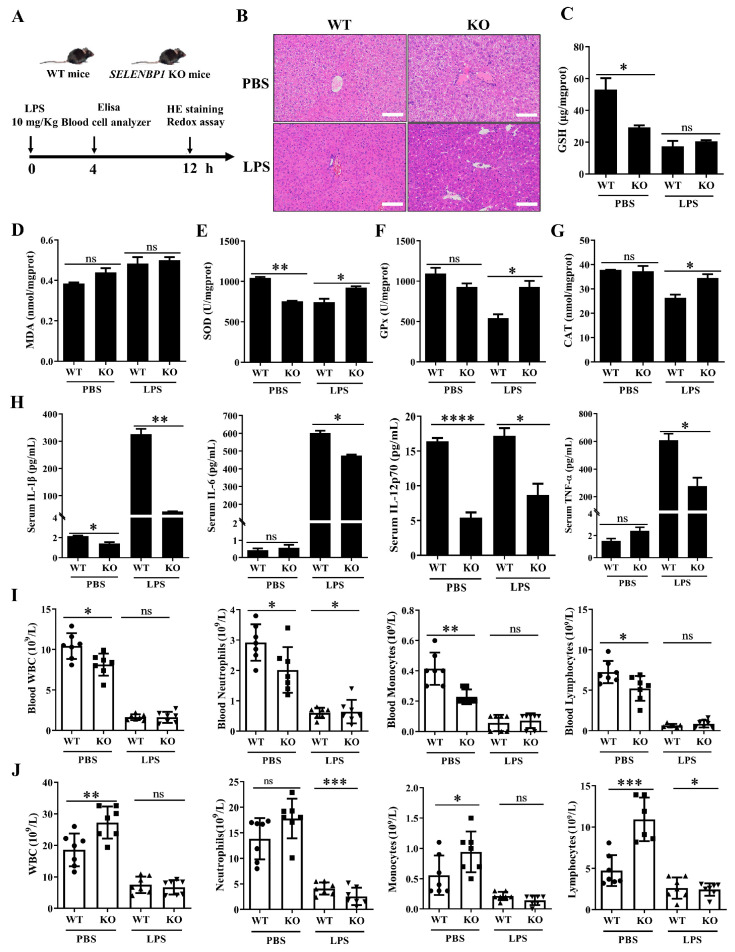
SELENBP1 deficiency alleviated liver injury in LPS-induced septic mice. (**A**) Illustration of septic mice. (**B**) Representative images of HE staining of the liver from septic mice (scale bar: 100 μm). The levels of GSH (**C**) and MDA (**D**), as well as the activities of SOD (**E**), GPx (**F**), and CAT (**G**), in the livers of septic mice were measured using corresponding kits (n = 6). (**H**) Serum levels of IL-1β, IL-6, IL-12, and TNF-α in septic mice were assayed using Elisa (n = 6). WBC, neutrophil, monocyte, and lymphocyte counts in the blood (**I**) and ascites (**J**) of septic mice were analyzed using a blood cell analyzer (n = 6 or 7). * *p* < 0.05; ** *p* < 0.01; *** *p* < 0.001; **** *p* < 0.0001; ns indicates no significance. GSH, glutathione; MDA, malondialdehyde; SOD, superoxide dismutase; GPx, glutathione peroxidase; CAT, catalase; IL, interleukin; TNF-α, tumor necrosis factor-α; WBC, white blood cells.

HE staining revealed that the liver structure of LPS-challenged *SELENBP1*-KO mice was clear, exhibiting hepatocyte edema, smaller hepatocyte septa, and minimal infiltration of inflammatory cell (Figure 3A,B). These findings indicate that *SELENBP1*-KO mice had minor liver damage. The redox status assessment indicated that LPS-challenged *SELENBP1*-KO mice exhibited enhanced antioxidant capability, including increased activities of SOD, GPx, and CAT (Figure 3C–G). Previous studies have found that elevated inflammation and immunosuppression resulted in a dysregulated response to infection [27,28,29,30]. Thus, both the inflammation and immunological status were evaluated. *SELENBP1*-KO mice treated with LPS showed lower levels of serum pro-inflammatory cytokines such as IL-1β, IL-6, IL-12, and TNF-α (Figure 3H). Furthermore, reduced WBC, neutrophil, monocyte, and lymphocyte counts were found in the blood of PBS-treated *SELENBP1*-KO mice, while increased WBC, monocyte, and lymphocyte counts were observed in the ascites. LPS-treated *SELENBP1*-KO mice had higher blood neutrophil levels, but lower ascites neutrophil and lymphocyte counts (Figure 3I,J). These findings indicated that the minor liver injury observed in *SELENBP1*-KO mice was associated with increased antioxidant capacity, decreased proinflammatory cytokines, and a dysregulated immunological microenvironment.

### 3.3. LPS-Challenged SELENBP1-KO Mice Showed Decreased Inflammation and Increased Immunosuppression in the Spleen

**Figure 4:**
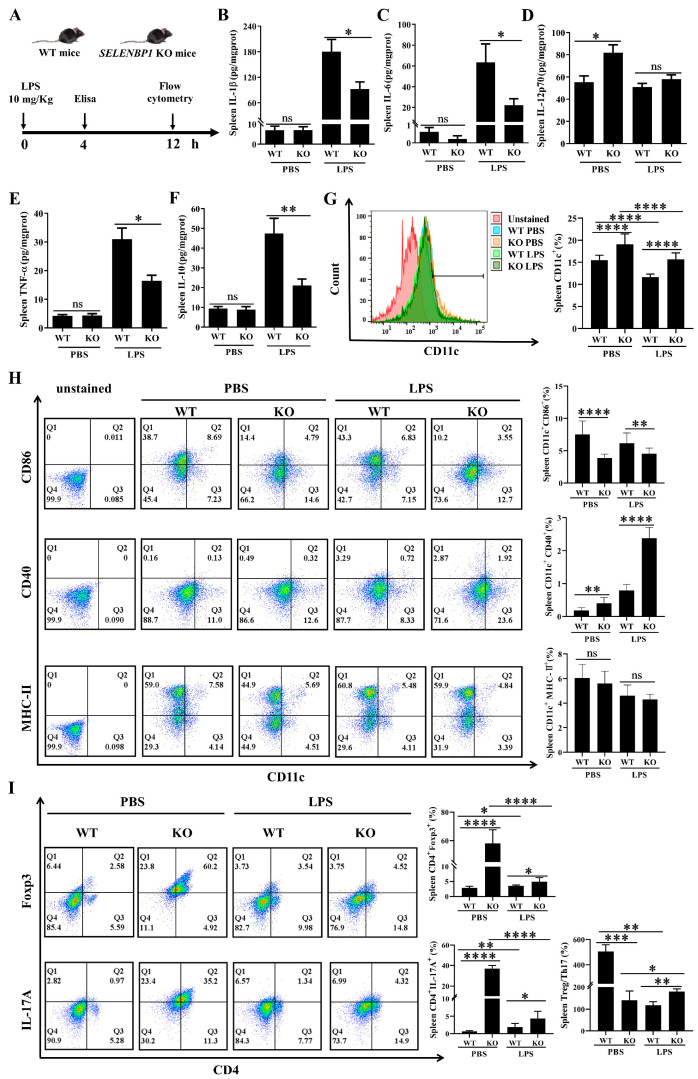
SELENBP1 deficiency reduced inflammation and increased immunosuppression in the spleens of septic mice. (**A**) Illustration of septic mice. Elisa was used to measure the levels of (**B**) IL-1β, (**C**) IL-6, (**D**) IL-12p70, (**E**) TNF-α, and (**F**) IL-10 in the spleen (n = 6). Flow cytometry was used to evaluate (**G**) CD11c^+^ cell numbers, (**H**) CD11c^+^CD86^+^, CD11c^+^CD40^+^, and CD11c^+^MHC-II^+^ DCs, as well as (**I**) CD4^+^Foxp3^+^ Tregs, CD4^+^IL-17A^+^ Th17 cells, and the Treg/Th17 ratio in the spleen (n = 6). * *p* < 0.05; ** *p* < 0.01; *** *p* < 0.001; **** *p* < 0.0001; ns indicates no significance. DCs, dendritic cells; Treg/Th17, regulatory T cell/T helper 17 cell.

Furthermore, we examined the inflammatory status of the spleen and found that LPS-treated *SELENBP1*-KO mice had lower pro-inflammatory cytokines (IL-1β, IL-6, and TNFα) and anti-inflammatory cytokine (IL-10) in the spleen (Figure 4A–F). Previous studies have suggested that DCs and DC-mediated Treg/Th17 imbalances play a crucial role in sepsis [28,31,35,49]. Consequently, we evaluated the markers of DCs, Treg, and Th17 in the spleen. The *SELENBP1*-KO mice demonstrated an increase in CD11c^+^ cell numbers regardless of LPS treatment (Figure 4G). Specifically, there was a reduction in CD11c^+^CD86^+^ DCs; however, there was an increase in CD11c^+^CD40^+^ DCs, CD4^+^Foxp3^+^ Tregs, and CD4^+^IL-17A^+^ Th17 cells. Furthermore, LPS-treated *SELENBP1*-KO mice displayed similar alterations in these cells but showed an elevated Treg/Th17 ratio (Figure 4H,I). This indicated that the decreased inflammatory status coupled with an increased Treg/Th17 ratio observed in the spleens of *SELENBP1*-KO mice contributed to their improved survival rates following LPS challenge.

### 3.4. SELENBP1 Deficiency Inhibited DCs Maturation

**Figure 5:**
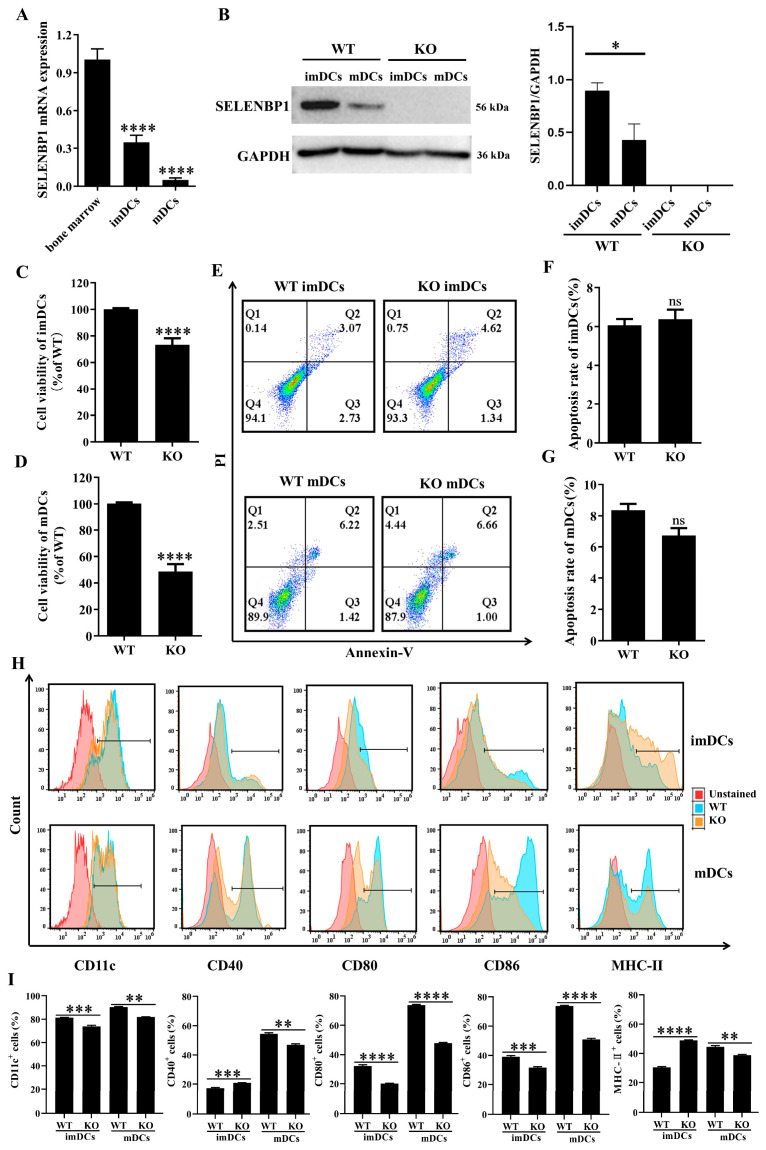
SELENBP1 deficiency impaired DCs maturation. (**A**) The levels of *SELENBP1* in bone marrow, imDCs, and mDCs were measured by RT-PCR (n = 3). (**B**) SELENBP1 protein levels in imDCs and mDCs derived from WT and *SELENBP1*-KO mice were detected using Western blotting (n = 3). (**C**,**D**) Cell viability and (**E**–**G**) apoptosis of imDCs and mDCs were tested using the CCK8 method and Annexin V-FITC/PI kit, respectively (n = 3). (**H**,**I**) Phenotypic markers of imDCs and mDCs were measured using flow cytometry and analyzed with FlowJo (n = 3). * *p* < 0.05; ** *p* < 0.01; *** *p* < 0.001; **** *p* < 0.0001; ns indicates no significance. imDCs, immature DCs; mDCs, mature DCs.

Based on previous findings, we hypothesized that SELENBP1-deficient DCs induced Treg/Th17 imbalance, contributing to the survival of LPS-induced septic mice [28,32,33,34]. First, the effect of *SELENBP1* KO on the maturation of bone marrow-derived DCs was investigated. *SELENBP1* expression gradually decreased during DCs differentiation and maturation, and SELENBP1 in bone marrow-derived DCs from *SELENBP1*-KO mice was absent (Figure 5A,B). Notably, *SELENBP1*-KO significantly reduced cell viability in DCs without affecting apoptosis (Figure 5C–G). Furthermore, *SELENBP1*-KO led to a significant decrease in the levels of CD11c, CD80, and CD86; conversely, CD40 and MHC-II levels were significantly increased in imDCs. In mDCs, there was a substantial reduction in these phenotypic markers (Figure 5H,I), leading to smDCs, which are also characteristic of tolDCs or DCregs. This demonstrated that *SELENBP1* KO impaired DCs’ maturation and drove their differentiation towards smDCs or tolDCs.

### 3.5. SELENBP1 Deficiency Altered the Immune Functions of DCs

**Figure 6:**
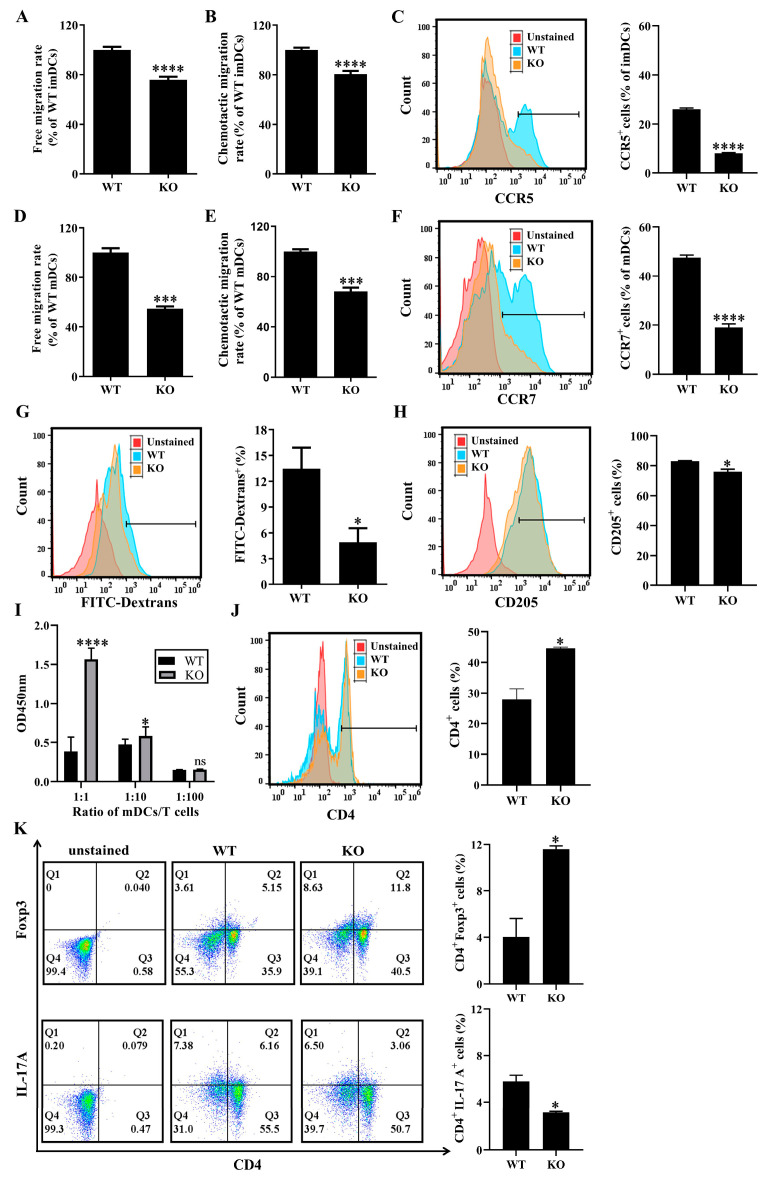
SELENBP1 deficiency altered the immune functions of DCs. The free and chemotactic migration of (**A**,**B**) imDCs and (**D**,**E**) mDCs were assayed using Transwell with or without chemokine, the chemokine receptors (**C**) CCR5 for imDCs and (**F**) CCR7 for mDCs were detected using flow cytometry (n = 3). (**G**,**H**) The phagocytic ability of imDCs to FITC-dextran and the phagocytic receptor CD205 were assayed using flow cytometry (n = 3). (**I**) The effect of mDCs on the proliferation of naïve CD4^+^ T cells was evaluated using the CCK8 method (n = 3), and (**J**) CD4^+^ T cells were counted using flow cytometry following co-culture with mDCs at a 1:1 ratio (n = 3). (**K**) CD4^+^Foxp3^+^ Tregs and CD4^+^IL-17A^+^ Th17 cells were detected using flow cytometry after co-culturing CD4^+^ T cells with mDCs at a ratio of 1:1 (n = 3). * *p* < 0.05; *** *p* < 0.001; **** *p* < 0.0001; ns indicates no significance.

Subsequently, the immunological functions of *SELENBP1*-KO DCs prepared from bone marrow were evaluated. Both free migration and chemokine-mediated migration were significantly reduced in SELENBP1-absent imDCs and mDCs (Figure 6A–F). Furthermore, CD205-mediated phagocytosis of dextrans was markedly diminished in imDCs (Figure 6G,H). Additionally, mDCs promoted the proliferation of naïve CD4^+^ T cells, resulting in an imbalance in the Treg/Th17 ratio characterized by an increase in CD4^+^Foxp3^+^ Tregs and a decrease in CD4^+^IL-17A^+^ Th17 cells (Figure 6I–K). This indicated that SELENBP1-deficient DCs functioned similarly to smDCs or tolDCs, contributing to Treg/Th17 imbalances and promoting immunological tolerance [32,33,34].

### 3.6. Redox Imbalance Involved the Immune Function of SELENBP1-Deficient DCs

**Figure 7:**
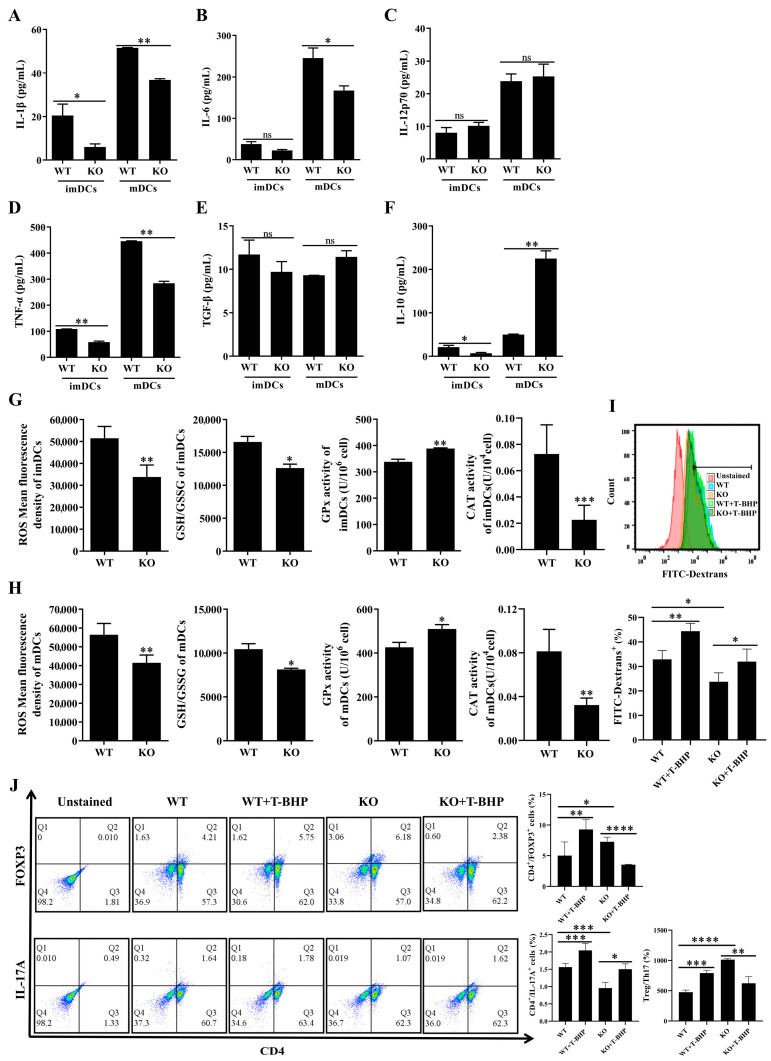
Redox imbalance involved the immune function of SELENBP1-deficient DCs. The levels of (**A**) IL-1β, (**B**) IL-6, (**C**) IL-12p70, (**D**) TNF-α, (**E**) TGF-β, and (**F**) IL-10 in DCs (n = 3) were measured using Elisa kits. ROS and GSH/GSSG levels, as well as the activities of GPx and CAT in the (**G**) imDCs and (**H**) mDCs were assayed using corresponding kits (n = 3). (**I**) The phagocytosis of imDCs to FITC-dextran was assayed using flow cytometry after T-BHP treatment (n = 9). (**J**) CD4^+^Foxp3^+^ Tregs and CD4^+^IL-17A^+^ Th17 cells were detected using flow cytometry after co-culturing CD4^+^ T cells with T-BHP-treated mDCs at a ratio of 1:1 (n = 9). * *p* < 0.05; ** *p* < 0.01; *** *p* < 0.001; **** *p* < 0.0001; ns indicates no significance. TGF-β, transforming growth factor-β; ROS, reactive oxygen species; GSSG, glutathione disulfide.

The aforementioned findings indicated that SELENBP1-deficient DCs exhibited a state of tolerance, resulting in a Treg/Th17 imbalance with altered phenotypic markers. It was previously shown that DCreg-induced tolerance was related to inflammatory cytokines [32]. Consequently, we investigated the impact of SELENBP1 deficiency on cytokine secretion in DCs. SELENBP1-KO mDCs had lower levels of IL-1β, IL-6, and TNFα while exhibiting elevated levels of IL-10 (Figure 7A–F), indicating that the Treg/Th17 imbalance induced by SELENBP1-deficient DCs was related to inflammatory cytokines. Oxidative stress has been shown to induce inflammation [27] and impact the immunological activity of DCs [36,37]; therefore, the redox state was investigated. SELENBP1 KO resulted in decreased ROS levels, as well as the GSH/GSSG ratio and CAT activity, while increasing GPx activity in imDCs and mDCs (Figure 7G,H). This suggested that redox imbalance may play a role in DCs immune function.

To further confirm the involvement of oxidative stress in SELENBP1-deficient DCs, we evaluated the immunological activity of *SELENBP1*-KO DCs following treatment with T-BHP, a ROS donor. A non-cytotoxic dose of 90 μM was selected for this purpose; ROS supplementation restored ROS levels in *SELENBP1*-KO DCs (Supplementary Figure S2). Notably, ROS supplementation restored the phagocytosis of imDCs (Figure 7I). Moreover, it ameliorated CD4^+^ T cell proliferation induced by mDCs and the Treg/Th17 imbalance observed previously (Figure 7J, Supplementary Figure S3). The findings revealed that redox imbalance is involved in the immunological function of SELENBP1-deficient DCs.

### 3.7. Assessment of SELENBP1-KO mDCs for Sepsis

**Figure 8:**
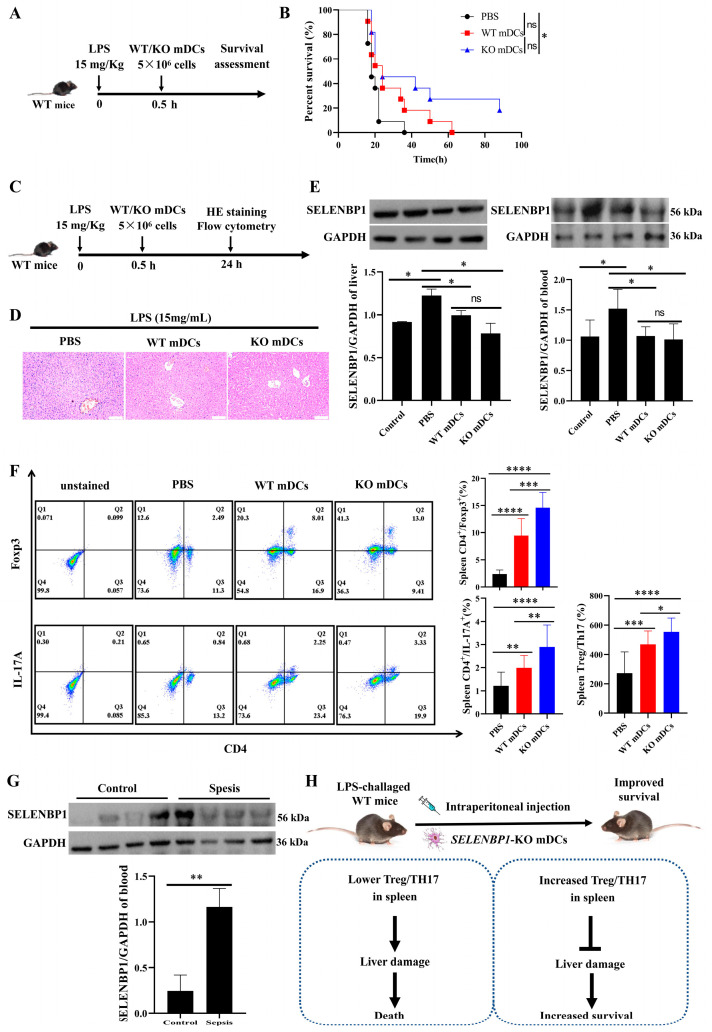
Assessment of SELENBP1-deficient mDCs for sepsis. (**A**) Illustration of SELENBP1-deficient mDCs treatment for WT-septic mice. (**B**) The survival time of sepsis-induced mice after treatment with PBS, WT, or *SELENBP1*-KO mDCs (n = 11). (**C**) Illustration of SELENBP1-deficient mDCs’ therapeutic effect for WT-septic mice. (**D**) Representative images of HE staining of the liver from septic mice after treatment with PBS, WT, or *SELENBP1*-KO mDCs (scale bar: 100 μm). (**E**) SELENBP1 levels in the liver (n = 3) and blood (n = 6) of septic mice after therapy. (**F**) CD4^+^Foxp3^+^ Tregs, CD4^+^IL-17A^+^ Th17 cells, and the Treg/Th17 ratio in the spleen from septic mice after treatment with PBS, WT, or *SELENBP1*-KO mDCs (n = 4). (**G**) SELENBP1 levels in the blood of septic patients and healthy controls (n = 4). (**H**) Schematic diagram of *SELENBP1*-KO mDCs improving survival in mice with sepsis. * *p* < 0.05; ** *p* < 0.01; *** *p* < 0.001; **** *p* < 0.0001; ns indicates no significance.

To evaluate the therapeutic potential of SELENBP1-deficient DCs in sepsis, bone marrow-derived mDCs from WT and KO mice were injected intraperitoneally into WT-septic mice (Figure 8A). The administration of *SELENBP1*-KO mDCs significantly prolonged the survival time of sepsis-induced mice (Figure 8B) and resulted in milder liver damage as indicated by histological analyses (Figure 8C,D). Notably, treatment with *SELENBP1*-KO mDCs led to a reduction in the LPS-induced SELENBP1 elevation observed in both the liver and blood of septic mice (Figure 8E, Supplementary Figure S1D). In addition, the Treg/Th17 ratio in the spleen was significantly increased (Figure 8F). Importantly, elevated levels of SELENBP1 were confirmed in clinical samples obtained from patients diagnosed with sepsis (Figure 8G).

**Figure 9:**
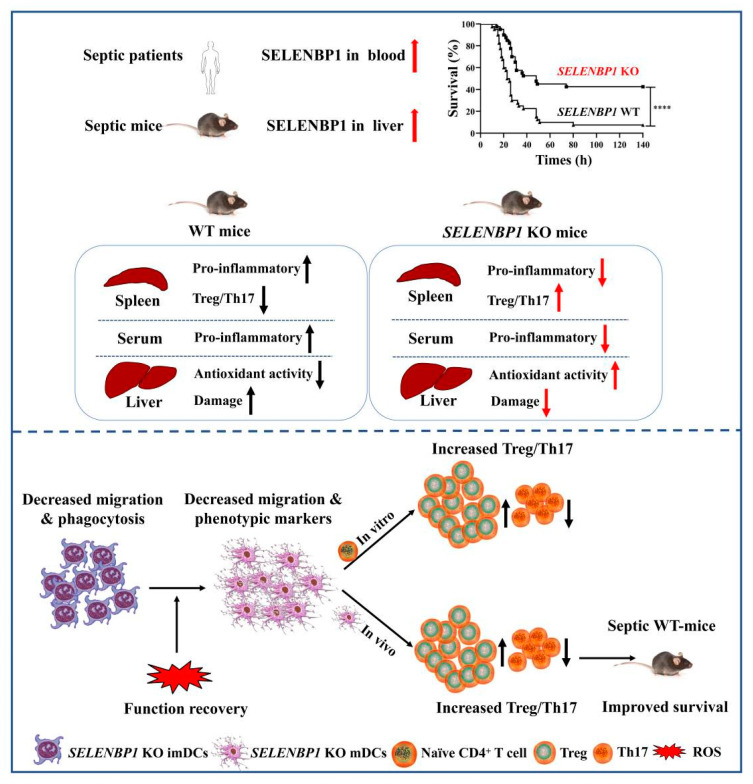
SELENBP1 is a biomarker of sepsis, and *SELENBP1*-KO mDCs prolonged the survival time of septic mice. **** *p* < 0.0001.

Collectively, these findings indicate that the Treg/Th17 imbalance induced by *SELENBP1*-KO mDCs was implicated in the regulation of liver injury during sepsis. Moreover, tolDCs resulting from SELENBP1 knockout could be a potential treatment for sepsis (Figure 8H). In summary, our results indicate that SELENBP1 serves as a biomarker for sepsis; its deficiency induces DCs into a tolerant state by regulating oxidative stress, leading to an increase in the proportion of Treg/Th17, which alleviates liver damage in septic mice (Figure 9).

## 4. Discussion

Sepsis is a serious disease with a high mortality rate, and advances have been made in the identification, prognosis, and treatment of this disease. Numerous biomarkers have been discovered based on clinical observations and machine learning, including SELENOP. However, the functions of most biomarkers remain unclear due to the complexity of sepsis [50,51]. SELENBP1 was recently identified as a biomarker of kidney injury [10], with elevated levels of SELENBP1 in urine from septic patients potentially indicating renal injury [26]. However, the role of SELENBP1 in sepsis remains unknown.

In this study, we observed that SELENBP1 levels were increased in both septic human blood and the mouse liver. Furthermore, LPS-challenged *SELENBP1*-KO mice had reduced liver inflammation and damage while demonstrating improved survival rates. This may be attributed to an enhancement in DC-mediated Treg/Th17 responses alongside decreased inflammation within the spleen. Consistently, an increase in GPX activity was noted in the livers of LPS-treated *SELENBP1*-KO mice; this elevation may be related to GPX1 [6,19]. However, opposing oxidative stress in the hippocampus and liver of *SELENBP1*-KO mice may be associated with different models and tissue specificities [19]. Targeting oxidative stress has shown promise in alleviating tissue damage and acute inflammation resulting from sepsis [27], as evidenced by findings related to the livers and spleens of LPS-induced *SELENBP1*-KO mice. Additionally, a high Treg/Th17 ratio predicted a positive prognosis in sepsis [28,31], which was similar to the findings observed within the spleens of LPS-challenged *SELENBP1*-KO mice. These findings suggested that SELENBP1 plays a critical regulatory role concerning oxidative stress, inflammation, and immune functions during sepsis.

Previous studies have revealed the essential role of neutrophils, monocytes, and lymphocytes in sepsis [28,29,30,52,53,54], with variations in their quantities observed in this study. The particular relevance of these alterations requires further investigation. We discovered that *SELENBP1*-KO reduced ascites neutrophils in septic mice, consistent with findings that a lower neutrophil/monocyte ratio indicates a positive prognosis [54]. Furthermore, macrophages play a crucial role in the liver damage caused by elevated levels of IL-1β and TNF-α both in the liver and serum of LPS-induced sepsis mice [55]. Consistently, *SELENBP1*-KO mice had lower levels of IL-1β and TNF-α in their spleen and serum, leading to less liver damage.

DCs, as a link between innate and adaptive immunity, play a key role in immune suppression during sepsis [28,29,30,52,53,54]. Thus, we primarily focused on DCs in LPS-challenged *SELENBP1*-KO mice. Our results revealed a decrease in CD11c^+^CD86^+^ DCs and an increase in CD11c^+^CD40^+^ DCs. To further clarify the immunological activities of SELENBP1, bone marrow-derived DCs were prepared for examination. We observed that SELENBP1 deficiency impaired DC maturation and reduced the levels of CD40, CD80, CD86, and MHC-II, indicating a tolerance phenotype [32,33]. Moreover, DC-secreted IL1β, IL-6, and TNFα promote Th17 development, while IL10 secreted by DCs promotes Treg cell differentiation [32,56]. This study found that SELENBP1 deficiency reduced levels of IL-1β, IL-6, and TNFα while increasing levels of IL-10. These observations are consistent with cytokine levels typical for tolDCs [32,57], revealing an enhanced Treg/Th17 ratio in our investigation. Collectively these findings revealed that SELENBP1 deficiency promoted DCs into tolDCs. Previous research [19,20,21,22] and this study discovered that SELENBP1 is involved in oxidative stress, and ROS are necessary for DCs’ maturation [36,37]. Indeed, SELENBP1 deficiency decreased ROS levels and had a redox imbalance in DCs. Actually, ROS supplementation restored DC immunological activity, including phagocytosis of imDCs and mDC-induced Treg/Th17 imbalance. Thus, we suggested that SELENBP1 deletion induced DCs’ differentiation into tolDCs through a reduction in ROS.

Studies have shown that monitoring immune homeostasis and providing personalized immunomodulatory therapy are critical for improving survival in patients with sepsis [28,29,30,58]. Our present study discovered that the enhanced survival rate of sepsis mice with *SELENBP1* deletion was associated with a higher Treg/Th17 ratio. In vitro investigations revealed that *SELENBP1*-KO mDCs exhibiting tolDCs features could modulate the Treg/Th17 ratio. Therefore, we propose that *SELENBP1*-KO mDCs may be utilized as a therapeutic strategy for treating sepsis. Indeed, treatment with *SELENBP1*-KO mDCs mitigated liver damage and extended lifespan in septic mice by increasing the Treg/Th17 ratio in the spleen. Furthermore, inducing tolDCs’ formation to promote immune tolerance has been proven safe and effective in treating animal models of autoimmune disorders. In addition, autoimmune diseases reduced the mortality risk in patients with sepsis [59,60,61]. Interestingly, transcriptomic analyses revealed that *SELENBP1*-KO mice exhibited molecular characteristics of colitis (unpublished), which may also contribute to the elevated survival rate seen in *SELENBP1*-KO septic mice [61]. Notably, we discovered that LPS-induced SELENBP1 increases in septic mice’s blood and liver were considerably decreased following treatment with *SELENBP1*-KO mDCs. Although treatment with WT mDCs reduced the SELENBP1 level, increased the Treg/Th17 ratio, and prolonged the survival time of sepsis mice, the therapeutic effect was not satisfactory. These findings suggest that SELENBP1 antibodies could offer potential benefits for treating sepsis; however, further research is needed.

Numerous studies have revealed that nanoselenium modulates immune cell activity by increasing selenoprotein levels, making it a potential therapeutic agent for conditions such as viral pneumonia [62,63], colitis [64], heart disease [65,66], ischemia/reperfusion damage [67], cancer [68,69,70], and other diseases. In addition, inhibiting selenoprotein activity is also a targeted treatment strategy for certain types of cancer [71]. Thus, it is worth investigating whether SELENBP1 impacts selenium metabolism and disrupts the expression of selenoproteins, hence influencing the course of sepsis. Our results suggested that SELENBP1 deficiency regulates DC maturation homeostasis and DC-mediated Treg balance, which could potentially be used in the treatment of sepsis, autoimmune diseases, and allergic diseases; however, more research is necessary to elucidate the optimal route of administration, time, concentration, and frequency of DC administration [60,72]. Additionally, it warrants investigation whether SELENBP1 deficiency affects the immune function of macrophages and T cells, especially the different subtypes of DCs [72]. Moreover, increased expression of SELENBP1 in glomerular mesangial cells during nephropathy has been shown to promote inflammation [73], aligning with observations regarding enhanced liver inflammation in our current study. Conversely, decreased SELENBP1 contributes to the risk of pulmonary arterial hypertension [74]. Therefore, the role of SELENBP1 in different tissues during sepsis requires further exploration.

## 5. Conclusions

The current in vivo investigation revealed that the prolonged survival of *SELENBP1*-KO septic mice was related to an enhanced antioxidant capacity in the liver, reduced pro-inflammatory levels in the blood, as well as decreased inflammation and an elevated Treg/Th17 ratio in the spleen. Furthermore, an in vitro study revealed that SELENBP1 deficiency induced a redox imbalance that inhibited DC maturation, resulting in a tolDC phenotype and an increase in the Treg/Th17 ratio. Additionally, *SELENBP1*-KO mDCs alleviated liver injury by increasing the Treg/Th17 ratio within the spleen, thereby enhancing the survival of sepsis mice. In summary, our study provides novel insights into how SELENBP1 is implicated in the progression of sepsis via its modulation of DC immunological activity, which may provide a novel way for effective treatment strategies against sepsis.

## Data Availability

Data are contained within this article.

## Supplementary Materials

The following supporting information can be downloaded at: https://www.mdpi.com/article/10.3390/antiox14040468/s1, Figure S1: (A) SELENBP1 protein levels in the liver, kidney, lung, and spleen of mice were detected using Western blotting (n = 3). (B) SELENBP1 protein levels in the livers of mice with LPS-induced sepsis (n = 3). (C) *SELENBP1* KO mice were generated and verified using proteins of the kidney (n = 3). (D) SELENBP1 levels in the liver of septic mice after therapy (n = 3); Figure S2: (A) The effect of T-BHP on the cell viability of imDCs and mDCs was detected by CCK8 kit (n = 9). (B) The H_2_O_2_ levels of imDCs and mDCs were detected by ROS GreenTM H_2_O_2_ Probe after with or without T-BHP treatment, and then evaluated using flow cytometry (n = 9); Figure S3: CD4^+^ T cells were counted using flow cytometry following co-culture with T-BHP treated-mDCs at a 1:1 ratio (n = 9).
