# Supplementary material for: Selenium-Binding Protein 1-Deficient Dendritic Cells Protect Mice from Sepsis by Increased Treg/Th17
Source: Antioxidants (Basel). 2025 Apr 14;14(4):468. doi: 10.3390/antiox14040468 (PMC12024190; doi:10.3390/antiox14040468)
Supplement: Supplementary file 1 [file antioxidants-14-00468-s001.zip › antioxidants-3537079-supplementary.pdf]

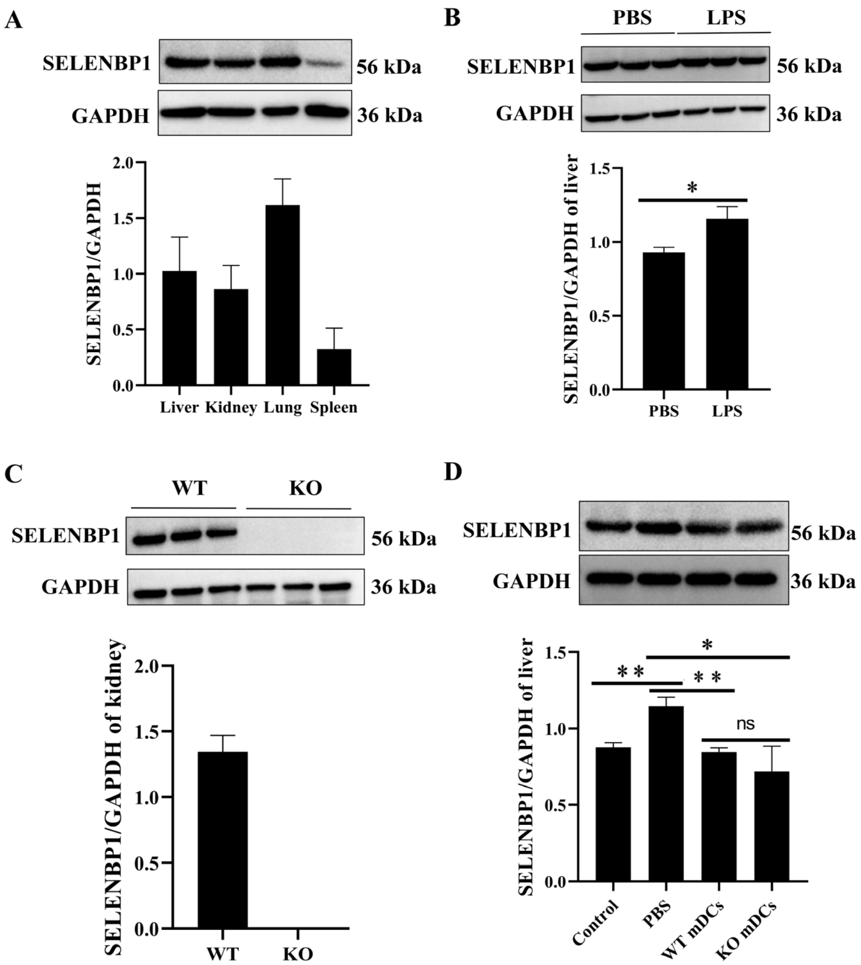

Figure S1. **(A)** SELENBP1 protein levels in the liver, kidney, lung, and spleen of mice were detected using Western blotting (n = 3). **(B)** SELENBP1 protein levels in the livers of mice with LPS-induced sepsis (n = 3). **(C)** *SELENBP1* KO mice were generated and verified using proteins of the kidney (n = 3). **(D)** SELENBP1 levels in the liver of septic mice after therapy (n = 3). $*p < 0.05$; $**p < 0.01$; ns, no significance.

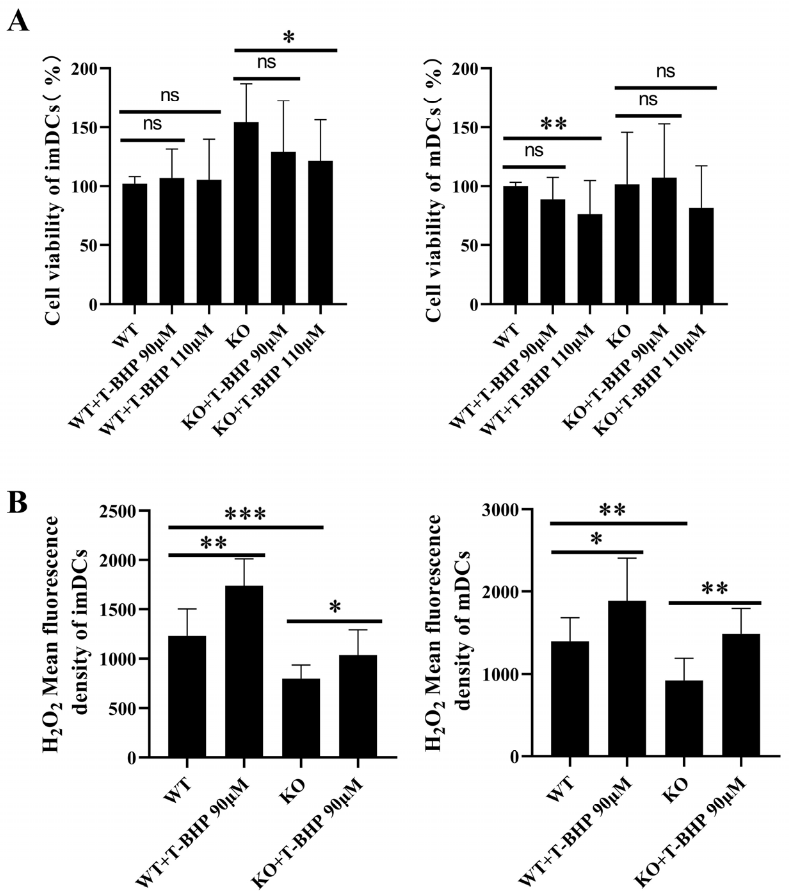

Figure S2. **(A)** The effect of T-BHP on the cell viability of imDCs and mDCs was detected by CCK8 kit (n = 9). **(B)** The H_2O_2 levels of imDCs and mDCs were detected by a ROS GreenTM H_2O_2 Probe after T-BHP treatment and then evaluated using flow cytometry (n = 9). *$p < 0.05$; **$p < 0.01$; ***$p < 0.001$; ns, no significance.

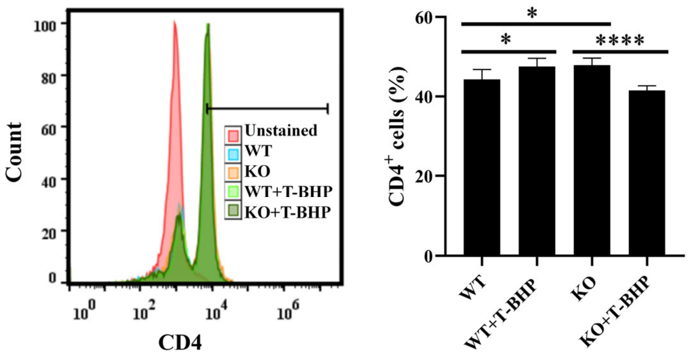

Figure S3. $CD4^+$ T cells were counted using flow cytometry following co-culture with T-BHP treated-mDCs at a 1:1 ratio (n = 9). **p* < 0.05; *****p* < 0.0001.
